# Curcumin inhibits activation induced by urban particulate material or titanium dioxide nanoparticles in primary human endothelial cells

**DOI:** 10.1371/journal.pone.0188169

**Published:** 2017-12-15

**Authors:** Angélica Montiel-Dávalos, Guadalupe Jazmin Silva Sánchez, Elizabeth Huerta-García, Cristhiam Rueda-Romero, Giovanny Soca Chafre, Irma B. Mitre-Aguilar, Ernesto Alfaro-Moreno, José Pedraza-Chaverri, Rebeca López-Marure

**Affiliations:** 1 Subdirección de Investigación Básica, Instituto Nacional de Cancerología, Ciudad de México, México; 2 Departamento de Fisiología (Biología Celular), Instituto Nacional de Cardiología "Ignacio Chávez", Ciudad de México, México; 3 Unidad de Bioquímica, Instituto Nacional de Ciencias Médicas y Nutrición Salvador Zubirán (INCMNSZ), Ciudad de México, México; 4 Swetox, Karolinska Institutet, Unit of Toxicology Sciences, Forskargatan, SE Södertälje, Sweden; 5 Departamento de Biología, Facultad de Química, Universidad Nacional Autónoma de México (UNAM), Ciudad de México, México; National Institutes of Health, UNITED STATES

## Abstract

Curcumin has protective effects against toxic agents and shows preventive properties for various diseases. Particulate material with an aerodynamic diameter of ≤10 μm (PM_10_) and titanium dioxide nanoparticles (TiO_2_-NPs) induce endothelial dysfunction and activation. We explored whether curcumin is able to attenuate different events related to endothelial activation. This includes adhesion, expression of adhesion molecules and oxidative stress induced by PM_10_ and TiO_2_-NPs. Human umbilical vein endothelial cells (HUVEC) were treated with 1, 10 and 100 μM curcumin for 1 h and then exposed to PM_10_ at 3 μg/cm^2^ or TiO_2_-NPs at 10 μg/cm^2^. Cell adhesion was evaluated by co-culture with U937 human myelomonocytic cells. Adhesion molecules expression was measured by flow cytometry after 3 or 24 h of exposure. Oxidative stress was determined by 2,7-dichlorodihydrofluorescein (H_2_DCF) oxidation. PM_10_ and TiO_2_-NPs induced the adhesion of U937 cells and the expression of E- and P-selectins, intercellular adhesion molecule-1 (ICAM-1), vascular cell adhesion molecule-1 (VCAM-1) and platelet-endothelial cell adhesion molecule-1 (PECAM-1). The expression of E- and P-selectins matched the adhesion of monocytes to HUVEC after 3 h. In HUVEC treated with 1 or 10 μM curcumin, the expression of adhesion molecules and monocytes adhesion was significantly diminished. Curcumin also partially reduced the H_2_DCF oxidation induced by PM_10_ and TiO_2_-NPs. Our results suggest an anti-inflammatory and antioxidant role by curcumin attenuating the activation caused on endothelial cells by exposure to particles. Therefore, curcumin could be useful in the treatment of diseases where an inflammatory process and endothelial activation are involved.

## Introduction

Curcumin is a phenolic antioxidant extracted from the rhizome of *Curcuma longa*. It is commonly used as a condiment in food (part of curry), as additive and colorant in the food industry, and as a natural pigment in cosmetics and textile industry. In traditional medicine of India and China, curcumin is considered a therapeutic agent for various diseases. Curcumin has different biological functions, particularly anti-inflammatory [1], antimicrobial [2], anticancer [3], neuroprotective [4], hepatoprotective [5], cardioprotective [6] and renoprotective [7, 8].

Curcumin is a bifunctional antioxidant that interacts with reactive species, stabilizes molecules and induces the expression of various cytoprotective and antioxidant proteins [9]. Curcumin is able to scavenge superoxide anion, hydrogen peroxide, singlet oxygen; nitric oxide, peroxynitrite anion and peroxyl radicals; hydroxyl radicals [10–15]. Together these effects could partly explain some of the cytoprotective effects of curcumin; coupled with its chemical structure, having functional groups such as β-diketone group [16], carbon-carbon double bonds and phenol rings with hydroxyl and methoxy groups [17]. Curcumin induces the nuclear factor (erythroid-derived 2)-like 2 factor (Nrf2) [18] and the expression of cytoprotective proteins such as superoxide dismutase, catalase, glutathione reductase, glutathione peroxidase, heme oxygenase 1, glutathione-S-transferase, the reduced form of nicotinamide adenine dinucleotide phosphate (NADPH), and quinone oxide reductase 1 [19].

Contrary to the effects induced by curcumin, particulate matter with an aerodynamic size of 10 nm (PM_10_), has an adverse effect on health. PM_10_ is a component of airborne particulate pollution in urban zones. It affects lung function, induces cancer [20, 21], increases the risk of allergies [22, 23], and has cardiovascular effects [24]. PM_10_ is associated with cell death by apoptosis and necrosis [25], an inflammatory response mediated by the secretion of cytokines, and DNA damage [26].

Similar toxic effects have been observed with some nanoparticles that range in size from 1–100 nm. Titanium dioxide nanoparticles (TiO_2_-NPs) are produced on large-scale in industry. They are used in the manufacture of cosmetics, sunscreens, pharmaceutical additives, drugs carriers, food colorants, microelectronics, and semiconductors, among others [27–29]. Due to their small size, they can penetrate basic biological structures affecting their function [30]. Also, TiO_2_-NPs can induce toxic effects on cardiac tissue [31], affecting cells of the circulatory system [32]. TiO_2_-NPs reduce cell viability and increase oxidative stress in several cell lines [33].

We have previously demonstrated that HUVEC exposed to PM_10_ or TiO_2_-NPs increased the expression of adhesion molecules and the adhesion of U937 cells inducing endothelial dysfunction and activation [34, 35]. The latter is characterized by the endothelial expression of early adhesion molecules such as endothelial leukocyte adhesion molecule (ELAM, also known as E-selectin) and P-selectin; and late cell-surface adhesion molecules such as vascular cell adhesion molecule 1 (VCAM-1), intercellular adhesion molecule 1 (ICAM-1), and platelet endothelial cell adhesion molecule 1 (PECAM-1) [36]. Endothelial cell activation is induced by proinflammatory cytokines, such as TNF-α and IL-6, among others and facilitates the recruitment and attachment of circulating leukocytes to the vessel wall. On the other hand, endothelial dysfunction is defined as the decreased synthesis, release, and/or activity of endothelium-derived nitric oxide (NO). Endothelial cell activation and dysfunction are linked because NO donors suppressed the expression of adhesion molecules and monocyte adhesion [37, 38]. Endothelial activation could clearly lead to endothelial dysfunction by inhibiting the endothelial nitric oxide synthase (eNOS) expression [39] and decreasing NO bioavailability through the induction of reactive oxygen species (ROS).

There is no evidence on curcumin's ability to protect endothelial cells and prevent endothelial activation induced by these particles, therefore, we explored whether curcumin is capable to attenuate cell adhesion, the expression of adhesion molecules and oxidative stress induced by PM_10_ and TiO_2_-NPs in HUVEC.

## Methods

### Materials

M199 and RPMI 1640 media, fetal bovine serum (FBS) and antibiotic-antimycotic mix (100X) were purchased from GIBCO/BRL (Grand Island, NY, USA); 0.25% trypsin and tryple express were acquired from INVITROGEN (Waltham, MA, USA. Sterile plastic material for tissue culture was obtained from NUNC (Rochester, NY, USA) and CORNING (Glendale, AZ, USA). Flow cytometry reagents were purchased from Becton Dickinson (Franklin Lakes, NJ, USA). Tumor necrosis factor alpha (TNF-α) was purchased from R&D Systems (Minneapolis, MN, USA). Thymidine [methyl-^3^H] was supplied by Perkin Elmer (Boston, MA, USA). 2,7-dichlorodihydrofluorescein diacetate (H_2_DCFDA) was purchased from Molecular Probes, Invitrogen (Carlsbad, CA, USA). Peroxidase-labeled monoclonal antibody against Von Willebrand factor and all the fluorescein isothiocyanate (FITC)-labeled monoclonal antibodies *vs*. human adhesion molecules were purchased from Santa Cruz Biotechnology (Santa Cruz, CA, USA). TiO_2_-NPs were acquired from Paris Drugstore (Mexico City, Mexico).

### PM_10_ and TiO_2_-NPs

PM_10_ were collected in downtown Mexico City in the period from 2004–2005 with a high-volume particle collector (GMW model 1200 VFC, Sierra Andersen) on a cellulose nitrate matrix, and processed as previously described [40]. 1 mg/mL suspensions were prepared immediately before cell exposure, and diluted as required.

TiO_2_-NPs were previously characterized by our work group [41], showing a mean nanoparticle size of 50 nm, the formation of aggregates with a mean size of 421 nm, a ζ-potential of -6.98 mV, and a BET surface area of 46.8 ± 1.6 m^2^/g.

### Cells and culture

Primary cultures of human umbilical vein endothelial cells (HUVEC) were obtained and cultured as described [42]. HUVEC phenotype was confirmed using Von Willebrand antigen staining. Cells exposed to 10 ng/mL human recombinant TNF-α and to 500 μM H_2_O_2_ to induce activation and oxidative stress, respectively, were the positive controls, and untreated cells were the negative controls. HUVEC were exposed to different concentrations of curcumin (1, 10, 100 μM), TiO_2_-NPs and PM_10_ (3 and 10 μg/cm^2^). Human leukemia pro-monocytic U937 cells were obtained from the American Type Culture Collection (ATCC® CRL-1593.2™). U937 cells were cultured in RPMI-1640 medium supplemented with 10% fetal bovine serum (FBS) and a mix of antibiotic-antimycotic.

### Adhesion of U937 cells to endothelial cells

Adhesion was evaluated as previously described by our group [37]. HUVEC were seeded at a density of 1 × 10^5^ cells/ml in 24-well tissue culture plates with 1 mL of supplemented M199 medium and treated with TNF-α, curcumin, TiO_2_-NPs and PM_10_ for 3 h; after this, U937 cells labeled with thymidine [methyl-^3^H] were added to the HUVEC culture for 3 h more. Finally, adhesion was measured by radioactivity in a scintillation counter (Tri-Carb 1600TR, Canberra-Packard, Meridien, CT, USA). Results were expressed as mean of count per minute (CPM) + standard error of the mean (SEM) of three experiments performed independently. CPM were directly proportional to U937 cells adhered to HUVEC.

### Adhesion molecules expression

The expression of early and late adhesion molecules was evaluated 3 and 24 h after exposure to TiO_2_-NPs or PM_10_, respectively. Cells were seeded in 24 well plates at a density of 1 × 10^4^ cells/well in M199 medium with serum withdrawal. Curcumin was added and cells were incubated for 1 h before the addition of TiO_2_-NPs or PM_10_. Cells were detached with 1 ml of Tryple Express solution for 5 min. Then they were incubated with different FITC-labeled monoclonal antibodies against human adhesion molecules diluted 1:20 for 1 h. This was followed by two washes with PBS-albumin (8% albumin and 0.02% sodium azide) and resuspension in 500 μl PBS. Cells were analyzed by flow cytometry using a Becton Dickinson FACSCalibur instrument (Franklin Lakes, NJ, USA). Results were normalized as the percentage of fluorescence compared with TNF-α-treated cells (positive control) considered as 100%. Fluorescence was calculated multiplying the FITC-positive cells number (FL1-H) by the mean of the fluorescence units (FU).

### Oxidative stress measure

Conversion of H_2_DCFDA into 2,7-(DCF) was used to assess oxidative stress and ROS production in HUVEC. Cells were incubated with H_2_DCFDA (10 μM) for 30 min at 37°C and washed twice with PBS. HUVEC were then cultured in the presence or absence of TiO_2_-NPs at 5 and 20 μg/cm^2^ for 1 h. H_2_O_2_ (500 μM) was used as a positive control to induce oxidative stress. After an extensive wash, fluorescence was measured by flow cytometry (Fascalibur, Becton Dickinson). Mean fluorescence intensity was calculated by multiplying the number of events (fluorescent cells) by the mean of the intensity presented by the Cell Quest software used for the analysis. Results were normalized as the percentage of H_2_DCF oxidation compared with H_2_O_2_-treated cells considered as 100%.

### Statistical analysis

All experiments were performed in duplicate, and at least three independent experiments were evaluated for each independent event or molecule that was measured. Data were expressed as mean (±SD) and comparisons between groups were made by an analysis of variance (ANOVA) test followed by Bonferroni’s post hoc test (Graph Pad Prism 5.0 for Mac OS X). A difference was considered statistically significant at p < 0.05.

## Results

### Curcumin inhibited the adhesion induced by PM_10_ and TiO_2_-NPs

**Fig 1:**
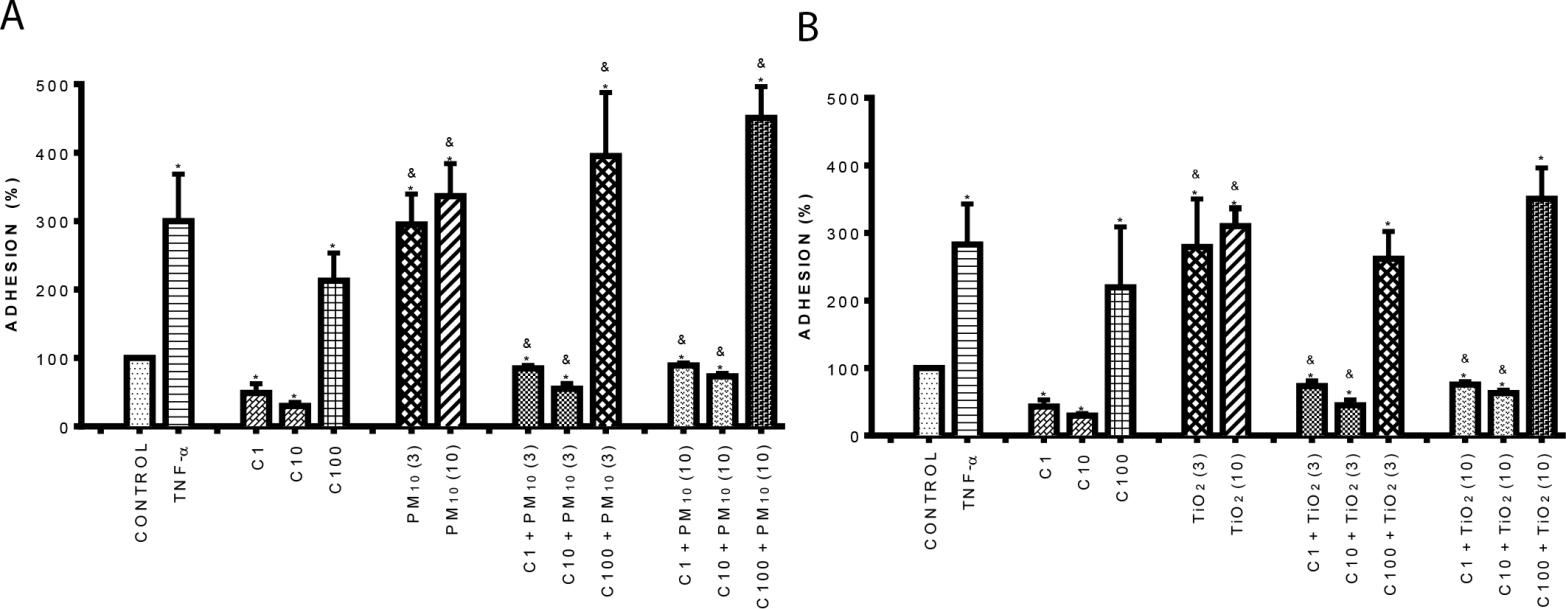
**Effect of curcumin on cell adhesion induced by PM**_**10**_ **(A) and TiO**_**2**_**-NPs (B)**. Cells were treated with curcumin at 1 (C1), 10 (C10) and 100 (C100) μM alone or in combination with 3 and 10 μg/cm^2^ of PM_10_ and TiO_2_-NPs (3) and (10) for 3 h. After this, U937 cells labeled with [^3^H]-thymidine were co-cultured with HUVEC for 3h more, and radioactivity in counts per minute was measured in a scintillation counter. TNF-α (10 ng/mL) was used as positive control. Curcumin was added 1 h before the addition of PM_10_ or TiO_2_-NPs. Data were expressed as percentage of adhesion with respect to positive control (100%) and shown as mean ± standard deviation (SD) of three separate experiments. p < 0.05, experiments compared with untreated cells (Control) (*) and with PM_10_ or TiO_2_-NPs alone (&).

We evaluated the adhesion of monocytes to endothelial cells, an important event during the inflammatory response. Our results showed that 1 and 10 μM curcumin did not have effect on adhesion of U937 cells to HUVEC (Fig 1); however, it was interesting that 100 μM curcumin increased adhesion by 120% compared to control (untreated cells). PM_10_ (Fig 1A) and TiO_2_-NPs (Fig 1B) increased adhesion by 2-fold compared with control (p < 0.05). Curcumin at 1 and 10 μM in combination with PM_10_ and TiO_2_-NPs reduced the adhesion induced by both particles at 3 and 10 μg/cm^2^, in comparison with particles alone; however, 100 μM curcumin was not able to produce the same effect (Fig 1A and 1B).

### Curcumin inhibited the expression of early adhesion molecules induced by PM_10_ and TiO_2_ NPs

**Fig 2:**
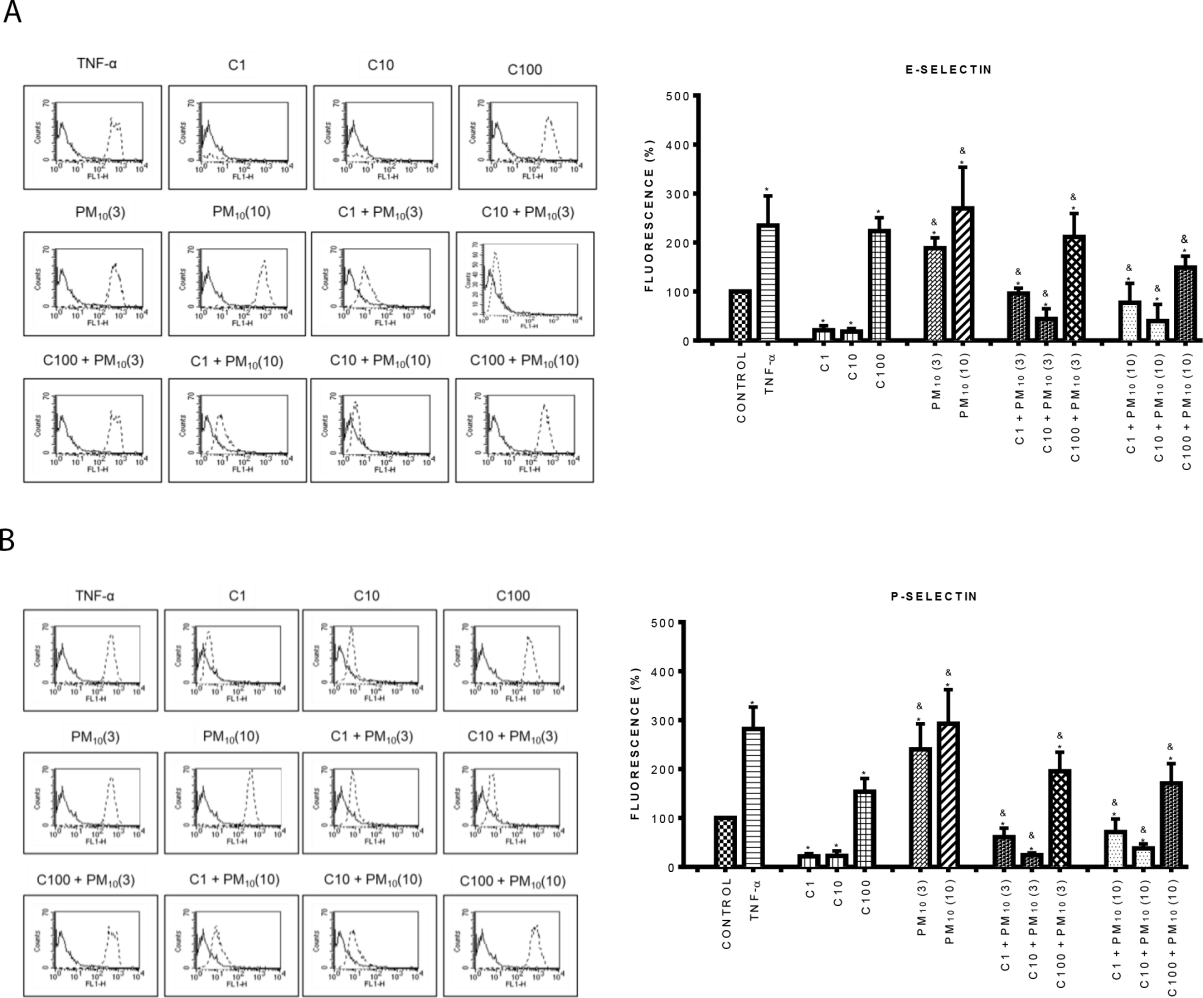
Effect of curcumin on the expression of early adhesion molecules induced by PM_10_. A) Top panel: E-selectin, B) Bottom panel: P-selectin. Cells were treated with curcumin at 1 (C1), 10 (C10) and 100 (C100) μM alone or in combination with 3 and 10 μg/cm^2^ of PM_10_ (3) and (10) for 3 h. The expression of early adhesion molecules was evaluated by flow cytometry. Curcumin was added 1 h before the addition of PM_10_. TNF-α (10 ng/mL) was used as positive control. The left side shows histograms of a representative experiment. Continuous lines correspond to control cells and dashed lines to treated cells. The right side shows data as mean ± standard deviation (SD) of three separate experiments, expressed as percentage of fluorescence in comparison with control (100%). p < 0.05, experiments compared with untreated cells (Control) (*) and with PM_10_ alone (&).

Cell adhesion in endothelial activation is mediated by adhesion molecules. We previously showed that PM_10_ and TiO_2_-NPs increased the expression of adhesion molecules in HUVEC [34, 35]. Therefore, in this work we determined the effect of curcumin on the expression of early adhesion molecules such as endothelial (E-) and platelet (P-) selectins induced by particles in HUVEC. Results showed that PM_10_ at 10 μg/cm^2^ induced the expression of E-selectin with a 170% increase in comparison with control (p < 0.05). Curcumin did not affect E-selectin expression at 1 and 10 μM, but at 100 μM it induced a strong increment, similar to that induced by PM_10_ alone (Fig 2A). PM_10_ plus curcumin at 1 and 10 μM concentration abolished E-selectin expression induced by particles roughly by 50 and 80%, respectively (Fig 2A). P-selectin expression was also strongly induced by PM_10_ (Fig 2B). The combination of curcumin with PM_10_ induced a significant decrease of P-selectin expression, compared with PM_10_ alone (p < 0.05); the greatest effect was observed at 10 μM curcumin, reaching levels lower that control (Fig 2B). It was interesting to observe that curcumin alone at 1 and 10 μM decreased the P-selectin expression in comparison with control cells.

**Fig 3:**
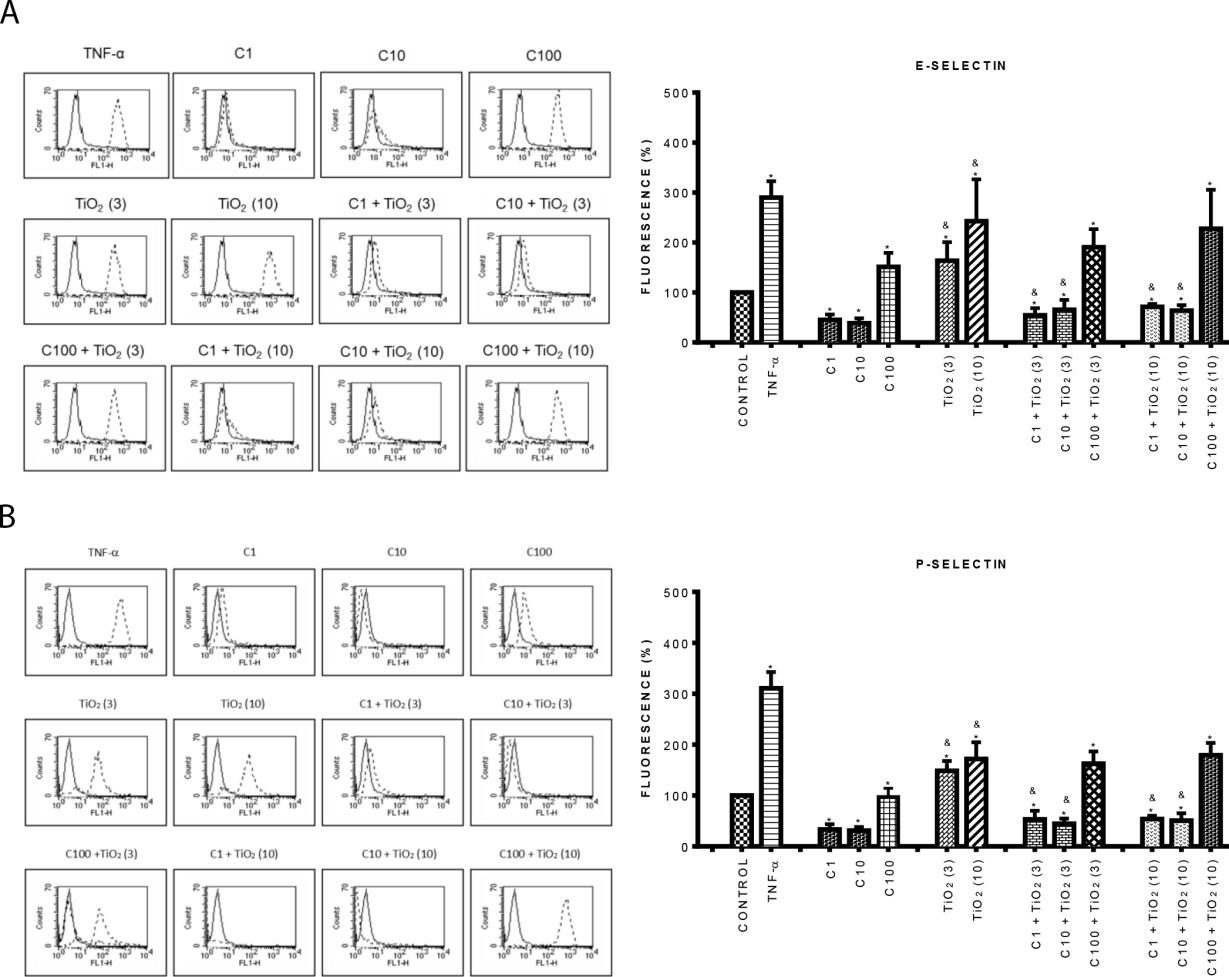
Effect of curcumin on the expression of early adhesion molecules induced by TiO_2_-NPs. A) Top panel: E-selectin, B) Bottom panel: P-selectin. Cells were treated with curcumin at 1 (C1), 10 (C10) and 100 (C100) μM alone or in combination with 3 and 10 μg/cm^2^ of TiO_2_-NPs (3) and (10) for 3 h. The expression of early adhesion molecules was evaluated by flow cytometry. Curcumin was added 1 h before the addition of TiO_2_-NPs. TNF- α (10 ng/mL) was used as positive control. The left side shows histograms of a representative experiment. Continuous lines correspond to control cells and dashed lines to treated cells. The right side shows data as mean ± standard deviation (SD) of three separate experiments, expressed as percentage of fluorescence in comparison with positive control (100%). p < 0.05, experiments compared with untreated cells (Control) (*) and with TiO_2_-NPs alone (&).

In relation to the effect of TiO_2_-NPs on E- and P-selectin expression, results showed that TiO_2_-NPs significantly increased E-selectin expression by 60% and 140% at 3 and 10 μg/cm^2^ compared to control, respectively (Fig 3A) (p < 0.05). When 1 and 10 μM curcumin was added in combination with nanoparticles, E-selectin expression decreased around 70% compared to nanoparticles alone, resembling the expression level of controls. Similar results were observed in P-selectin expression, where this decrease also reached the levels of untreated cells (control) (Fig 3B).

### Curcumin inhibited the expression of late adhesion molecules induced by PM_10_ and TiO_2_-NPs

**Fig 4:**
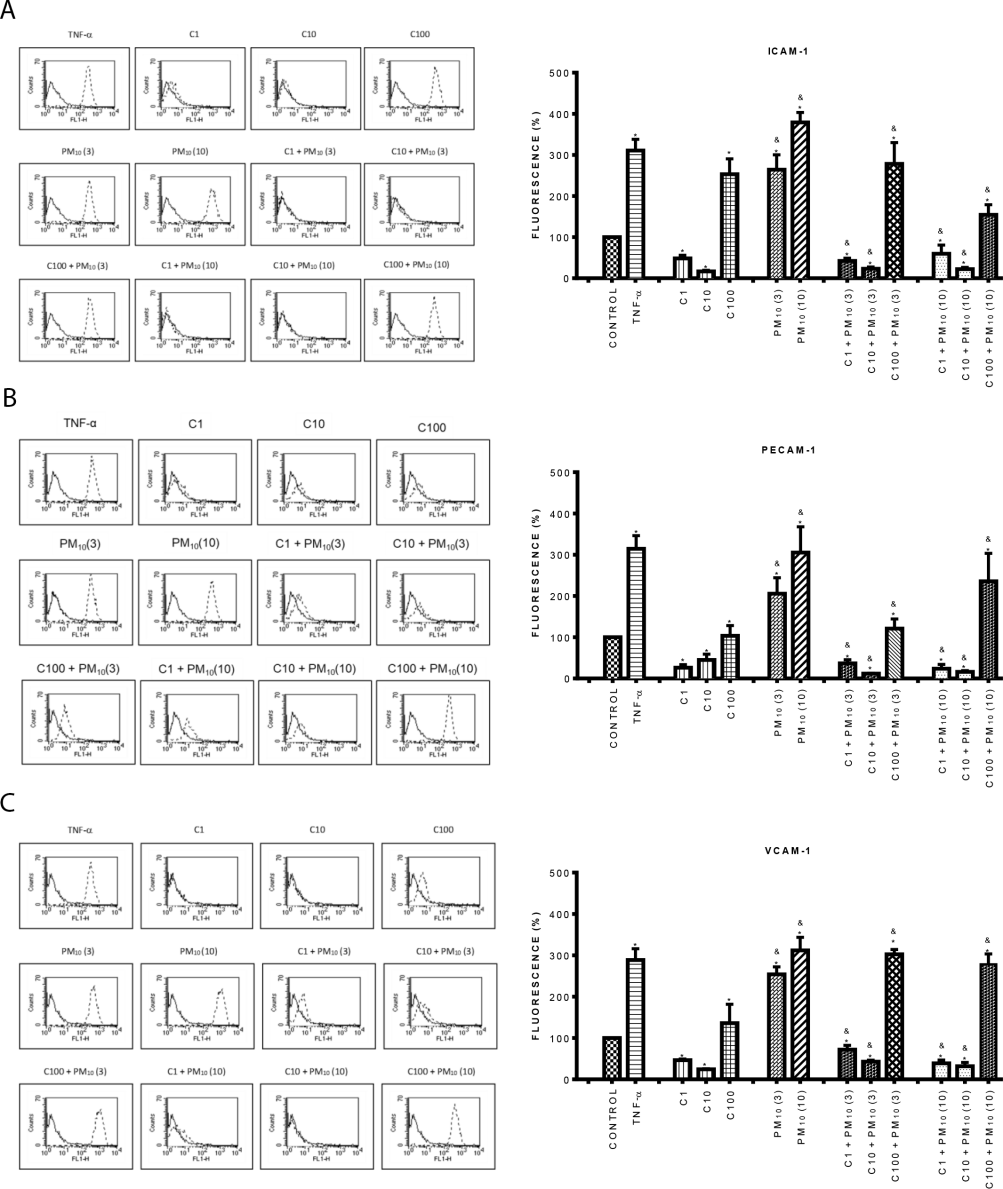
Effect of curcumin on the expression of late adhesion molecules induced by PM_10_. A) Top panel ICAM-1, B) Middle panel: PECAM-1, C) Bottom panel: VCAM-1. Cells were treated with curcumin at 1 (C1), 10 (C10) and 100 (C100) μM alone or in combination with 3 and 10 μg/cm^2^ of PM_10_ (3) and (10) for 24 h. The expression of late adhesion molecules was evaluated by flow cytometry. Curcumin was added 1 h before the addition of PM_10_. TNF-α (10 ng/mL) was used as positive control. The left side shows histograms of a representative experiment. Continuous lines correspond to control cells and dashed lines to treated cells. The right side shows data as mean ± standard deviation (SD) of three separate experiments, expressed as percentage of fluorescence in comparison with positive control (100%). p < 0.05, experiments compared with untreated cells (Control) (*) and with PM_10_ alone (&).

**Fig 5:**
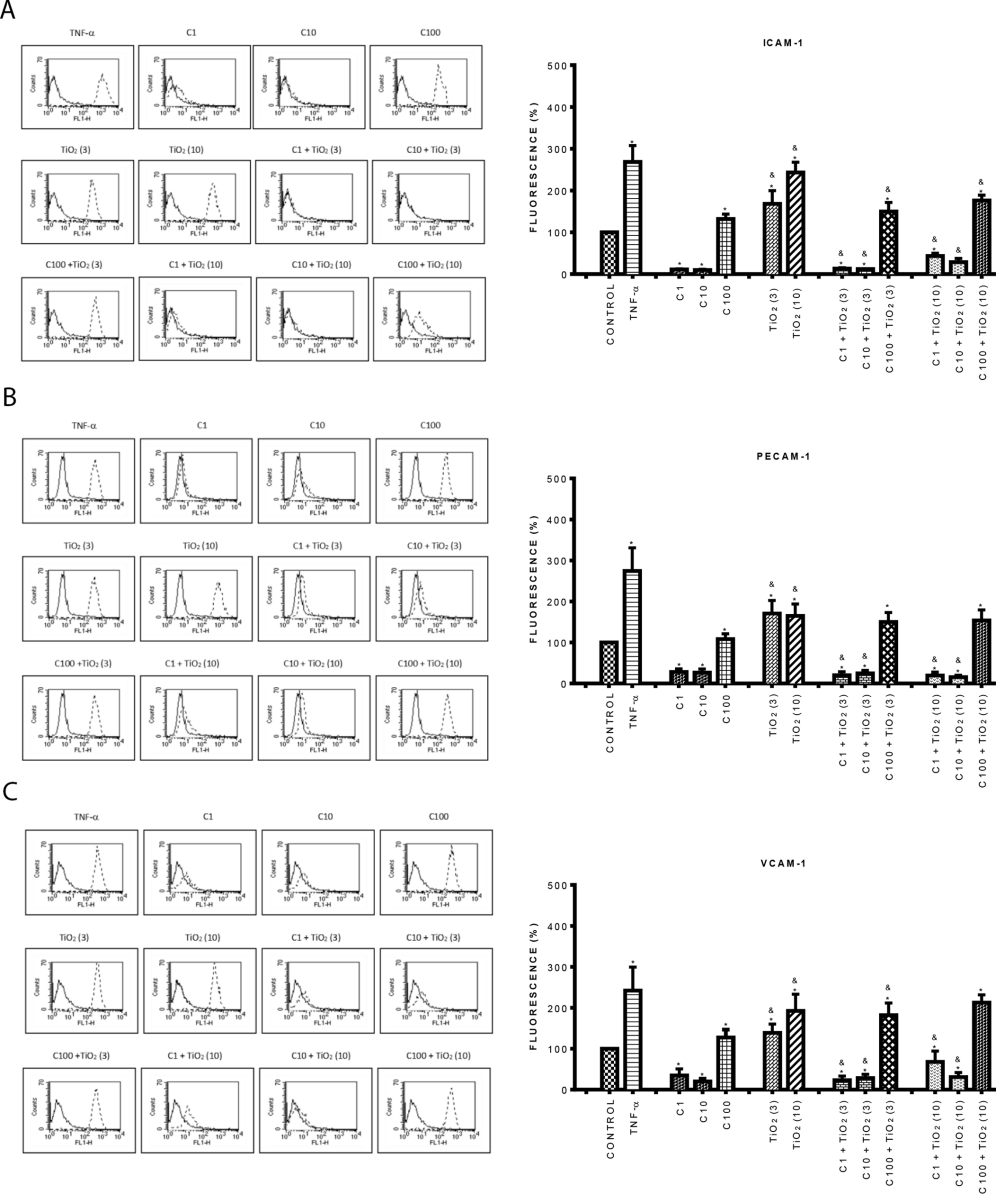
Effect of curcumin on the expression of late adhesion molecules induced by TiO_2_-NPs. A) Top panel ICAM-1, B) Middle panel: PECAM-1, C) Bottom panel: VCAM-1. Cells were treated with curcumin at 1 (C1), 10 (C10) and 100 (C100) μM alone or in combination with 3 and 10 μg/cm^2^ of TiO_2_-NPs (3) and (10) for 24 h. The expression of late adhesion molecules was evaluated by flow cytometry. Curcumin was added 1 h before the addition of TiO_2_-NPs. TNF-α (10 ng/mL) was used as positive control. The left side shows histograms of a representative experiment. The right side shows data as mean ± standard deviation (SD) of three separate experiments, expressed as percentage of fluorescence in comparison with positive control (100%). p < 0.05, experiments compared with untreated cells (Control) (*) and with TiO_2_-NPs alone (&).

Since curcumin inhibited the expression of early adhesion molecules, we also evaluated the expression of late adhesion molecules. Similar results to the previous ones were found with curcumin. At low concentrations (1, and 10 μM) curcumin reduced the expression of all the early and late adhesion molecules evaluated compared with control cells; while at a high concentration (100 μM), curcumin significantly increased their expression (Figs 4 and 5) (p < 0.05). PM_10_ at 3 μg/cm^2^ strongly increased the expression of ICAM-1 (160%) (Fig 4A), PECAM-1 (100%) (Fig 4B) and VCAM-1 (150%) (Fig 4C) compared to control cells, the greatest effect was observed at 10 μg/cm^2^. Endothelial cells treated with 1 and 10 μM curcumin plus PM_10_ (3 and 10 μ g/cm^2^) showed reduced expression of all these molecules reaching the control levels (Fig 4). Interestingly, we found that the combination of 100 μM curcumin plus 10 μg/cm^2^ PM_10_, decreased in 62% ICAM-1 expression in contrast to PM_10_ alone (Fig 4A).

On the other hand, TiO_2_-NPs at both concentrations significantly increased the expression of all late adhesion molecules by about 100% in comparison with control (Fig 5) (p < 0.05). The combination of curcumin (1 and 10 μM) plus TiO_2_-NPs (3 μg/cm^2^) completely abolished the increment induced by TiO_2_-NPs alone on all late adhesion molecules; however, 100 μM curcumin plus TiO_2_-NPs (10 μg/cm^2^), only partially decreased ICAM-1 expression compared with TiO_2_-NPs alone (Fig 5A).

### Curcumin inhibited the oxidative stress induced by PM_10_ and TiO_2_-NPs

**Fig 6:**
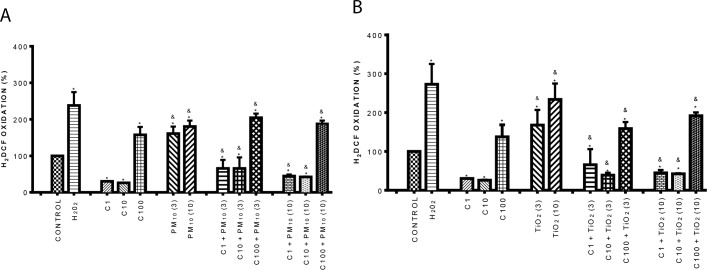
**Effect of curcumin on oxidative stress induced by PM**_**10**_ **(A) and TiO**_**2**_**-NPs (B)**. Cells were treated with curcumin at 1 (C1), 10 (C10) and 100 (C100) μM alone or in combination with 3 and 10 μg/cm^2^ of PM_10_ and TiO_2_-NPs (3) and (10) for 1 h. H_2_O_2_ (500 μM) was used as a positive control. Curcumin was added 1 h before the addition of PM_10_ or TiO_2_-NPs. Oxidative stress was evaluated by H_2_DCF oxidation by flow cytometry. Data were expressed as percentage of H_2_DCF oxidation with respect to control (100%) and shown as mean ± standard deviation (SD) of three separate experiments. p < 0.05, experiments compared with untreated cells (Control) (*) and with TiO_2_-NPs or PM_10_ alone (&).

Aberrant expression of inflammatory adhesion molecules is a consequence of ROS production [43], therefore we evaluated the levels of oxidative stress. Data showed that PM_10_ significantly increased H_2_DCF oxidation by 70% and 85%, at 3 and 10 μg/cm^2^ respectively compared to control. Curcumin decreased H_2_DCF oxidation in cells treated with 1 and 10 μM; however, the 100 μM concentration increased oxidation around 50%. In cells exposed to 1 and 10 μM curcumin in combination with PM_10_, the H_2_DCF oxidation decreased more that 60% in comparison with PM_10_ alone (Fig 6A).

On the other hand, TiO_2_-NPs significantly increased H_2_DCF oxidation by 70% and 145%, at 3 and 10 μg/cm^2^ respectively compared to control. Curcumin reduced H_2_DCF oxidation in cells treated with 1 and 10 μM compared with control; however, the 100 μM concentration strongly increased oxidation. In cells exposed to 1 and 10 μM curcumin in combination with TiO_2_-NPs, the H_2_DCF oxidation decreased by 62% and 80% in comparison with TiO_2_-NPs alone (Fig 6B).

## Discussion

In previous works, we have shown that TiO_2_-NPs and PM_10_ cause endothelial activation by stimulating the expression of adhesion molecules and adhesion of monocytic cells [34, 35, 41]. It has been shown that curcumin attenuates inflammatory responses of TNF-α-stimulated human endothelial cells [44]. In this work, we evaluated the ability of curcumin to inhibit some pro-inflammatory events induced by TiO_2_-NPs and PM_10_ on endothelial cells.

Our results showed that curcumin decreased the expression of early and late adhesion molecules on HUVEC exposed to TiO_2_-NPs and PM_10._ This was correlated with a reduced adhesion of U937 cells. These results are consistent with previous investigations showing that curcumin decreased U937 cells adhesion induced in HUVEC exposed to TNF-α [40]. Also, curcumin reduced pro-inflammatory effects of resistin in human endothelial cells, decreasing P-selectin expression, the levels of intracellular reactive oxygen species (ROS), NADPH activation and monocyte adhesion [45]. In renal epithelial NRK-52E cells, a curcumin analog (C66), significantly reduced overexpression of ICAM-1, VCAM-1, monocyte chemoattractant chemokine 1 (MCP-1), and macrophage adhesion [46]. In a murine model of sepsis, pre-treatment with curcumin modulated the adhesion of leukocytes and platelets in cerebral microcirculation [47]. In brain microvasculature endothelial cells, curcumin inhibited platelet adhesion [48]. In summary, it has been described that curcumin can regulate the expression of inflammatory cytokines, growth factors and receptors, enzymes, adhesion molecules and proteins related to apoptosis in different cellular models [49], strongly supporting the potent effect of curcumin against inflammatory events induced by damage produced by several agents.

Our results also showed that the effect of curcumin on the expression of adhesion molecules induced by nanoparticles and PMs was related with a decrease of oxidative stress, measured through H_2_DCF oxidation. It has been shown that a strong oxidative stress is involved in the pathophysiology of endothelial dysfunction, which accompanies a number of cardiovascular risk factors including hypercholesterolemia, hypertension, atherosclerosis, and diabetes. A similar protective effect of curcumin against oxidative stress was observed in the toxicity induced by 6-OHDA in dopaminergic neurons, where curcumin reduced ROS production [50]. Curcumin has been described as a strong antioxidant because it is a potent ROS scavenger, including O2●−, OH• and singlet oxygen, and may suppress ROS formation by upregulation of antioxidant enzymes [51]. In bovine endothelial cells, curcumin upregulates endothelial heme oxygenase-1 (HO-1) protein expression and increase heme oxygenase activity [52]. In addition to direct antioxidant activity, curcumin increases activities of antioxidant enzymes such as glutathione transferases and GPx inhibiting oxidative stress [53]. We think that curcumin could reduce oxidative stress induced by nanoparticles and PM_10_ by modulating the activity or expression of antioxidant enzymes in HUVEC.

It was very interesting to note that the protective effect induced by curcumin in this work was only observed at 1 and 10 μM; however, the 100 μM concentration had an opposite effect, increasing the expression of adhesion molecules, the adhesion of monocytes and H_2_DCF oxidation, similar to positive controls (TNF-α, H_2_O_2_) and particles. It was interesting to observe that like TNF-α positive controls, particles and curcumin (100 μM) which induced endothelial dysfunction and activation, also promoted morphological changes and decrease of cell proliferation (S1, S2 and S3 Figs), indicating toxicity. However, when curcumin at 10 μM was combined with both particles, these toxic effects were partially reversed. Previous works have reported a toxic effect of curcumin at high concentrations. Romero-Hernández *et al*. found that 100 μM curcumin reduces cell viability and induces morphological changes associated with a process called methuosis in four astrocytoma cell lines; however, the 10 μM concentration had no effect [54]. In other works, curcumin at low concentrations (< 20 μM) did not affect the viability of primary cultures of cerebellar granule neurons of rats, but at high concentrations (>30 μM), it altered the viability [4, 55]. Also, curcumin at 15 μM attenuates the increase in ROS production, the reduction of (GSH)/glutathione disulfide (GSSG) ratio, and cell death induced by hemin [4]. In rat liver epithelial cells, 5 and 10 μM curcumin reduces the toxic effects and ROS generation induced by iron [56]. Together, these results indicate that curcumin has a dual role, having a protective effect at low concentrations, and being toxic at high concentrations. The use of an *in vivo* model to study the effect of different curcumin concentrations will be useful, before its therapeutic application.

Many of the mechanisms related with curcumin effects remain unknown. The expression of adhesion molecules and oxidative stress are mediated by multiple intracellular signaling pathways such as mitogen-activated protein kinases (MAPK), phosphatidylinositol-3 kinase (PI3K)-Akt [57], the nuclear factor (NF-κB) pathway [58], among others. It will be very interesting to evaluate whether curcumin can modulate some of these pathways in HUVEC, which are important for the development of an inflammatory response.

## Conclusions

**Fig 7:**
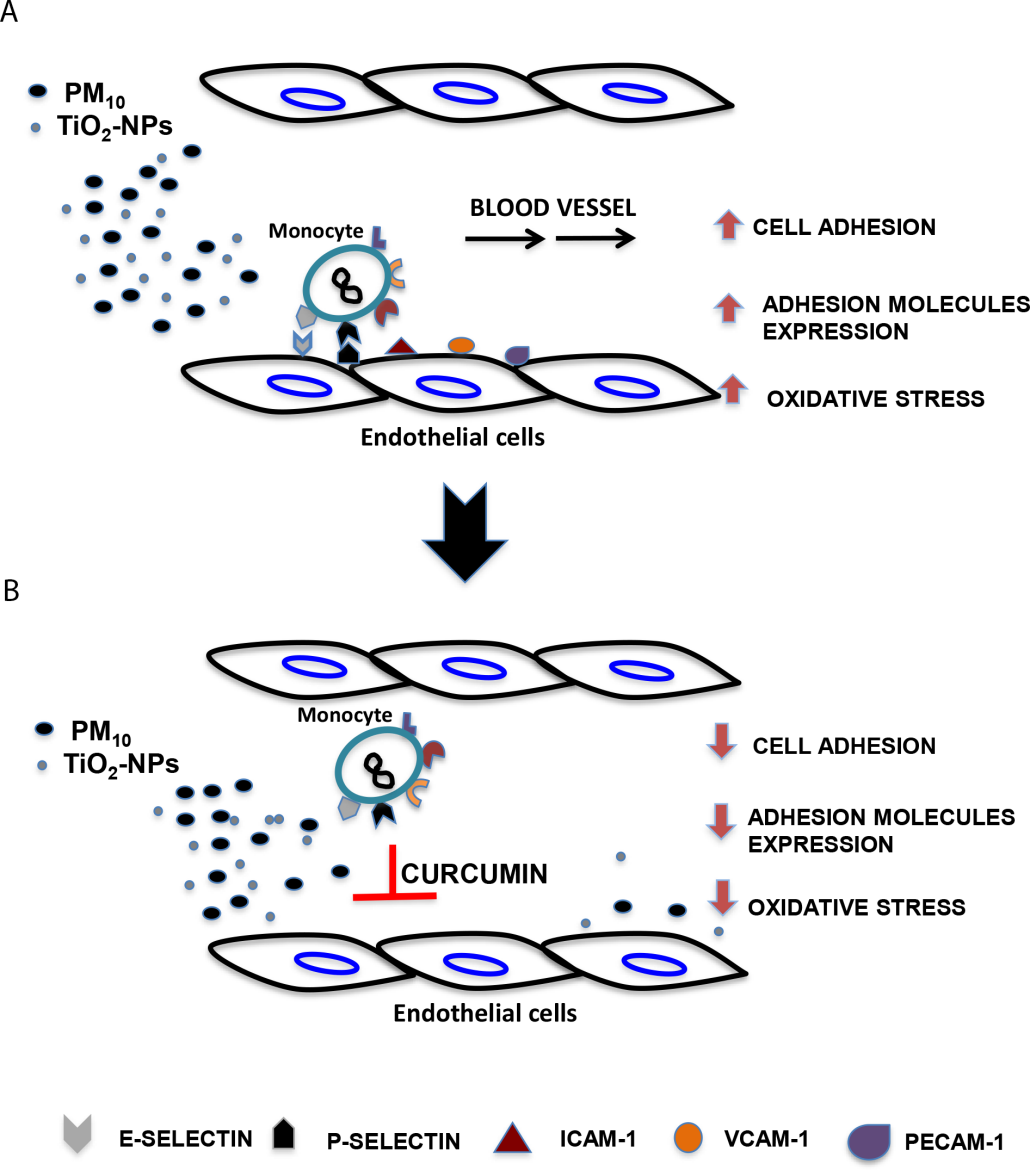
Curcumin abolished some pro-inflammatory events induced by nanoparticles and particulate matter in endothelial cells. Inflammatory events such as the increase of monocytes adhesion, the expression of early and late adhesion molecules and oxidative stress are induced in endothelial cells exposed to PMs and TiO_2_-NPs (A); however, pre-treatment with curcumin 1 h before the addition of particles, attenuate these events (B), indicating an anti-inflammatory and anti-oxidant role of curcumin.

Curcumin at 1 and 10 μM attenuates some pro-inflammatory events induced by nanoparticles and particulate matter in endothelial cells (Fig 7), suggesting that it could reduce inflammatory diseases derived from environmental pollution; however, more detailed studies are needed to corroborate the toxic effect of curcumin at high concentrations.

## Supporting information

S1 Fig Effect of curcumin on morphological changes induced by PM_10_. Cells were treated with curcumin at 1 (C1), 10 (C10) and 100 (C100) μM alone or in combination with 3 and 10 μg/cm^2^ of PM_10_ (3) and (10) for 24 h. Curcumin was added 1 h before the addition of PM_10_. TNF-α (10 ng/mL) was used as positive control. Photographs were taken with an optical microscope at 10X magnification.
(TIF)

S2 Fig Effect of curcumin on morphological changes induced by TiO_2_. Cells were treated with curcumin at 1 (C1), 10 (C10) and 100 (C100) μM alone or in combination with 3 and 10 μg/cm^2^ of TiO_2_-NPs (3) and (10) for 24 h. Curcumin was added 1 h before the addition of TiO_2_-NPs. TNF-α (10 ng/mL) was used as positive control. Photographs were taken with an optical microscope at 10X magnification.
(TIF)

S3 Fig Effect of curcumin on the inhibition of proliferation induced by PM_10_ and TiO_2_-NPs. Cells were treated with curcumin at 1 (C1), 10 (C10) and 100 (C100) μM alone or in combination with 3 and 10 μg/cm^2^ of PM_10_ (3) and (10) (A), and with 3 and 10 μg/cm^2^ of TiO_2_-NPs (3) and (10) (B) for 24 h. Proliferation was evaluated with crystal violet staining. Curcumin was added 1 h before the addition of PM_10_ and TiO_2_-NPs. TNF-α (10 ng/mL) was used as positive control. Data show the mean ± standard deviation (SD) of three separate experiments, expressed as percentage of proliferation compared to control (100%). p < 0.05, experiments compared with untreated cells (Control) (*) and with PM_10_ or TiO_2_-NPs alone (&).
(TIF)
