# Supplementary figures and images for: Curcumin inhibits activation induced by urban particulate material or titanium dioxide nanoparticles in primary human endothelial cells
Source: PLoS One. 2017 Dec 15;12(12):e0188169. doi: 10.1371/journal.pone.0188169 (PMC5731739; doi:10.1371/journal.pone.0188169)

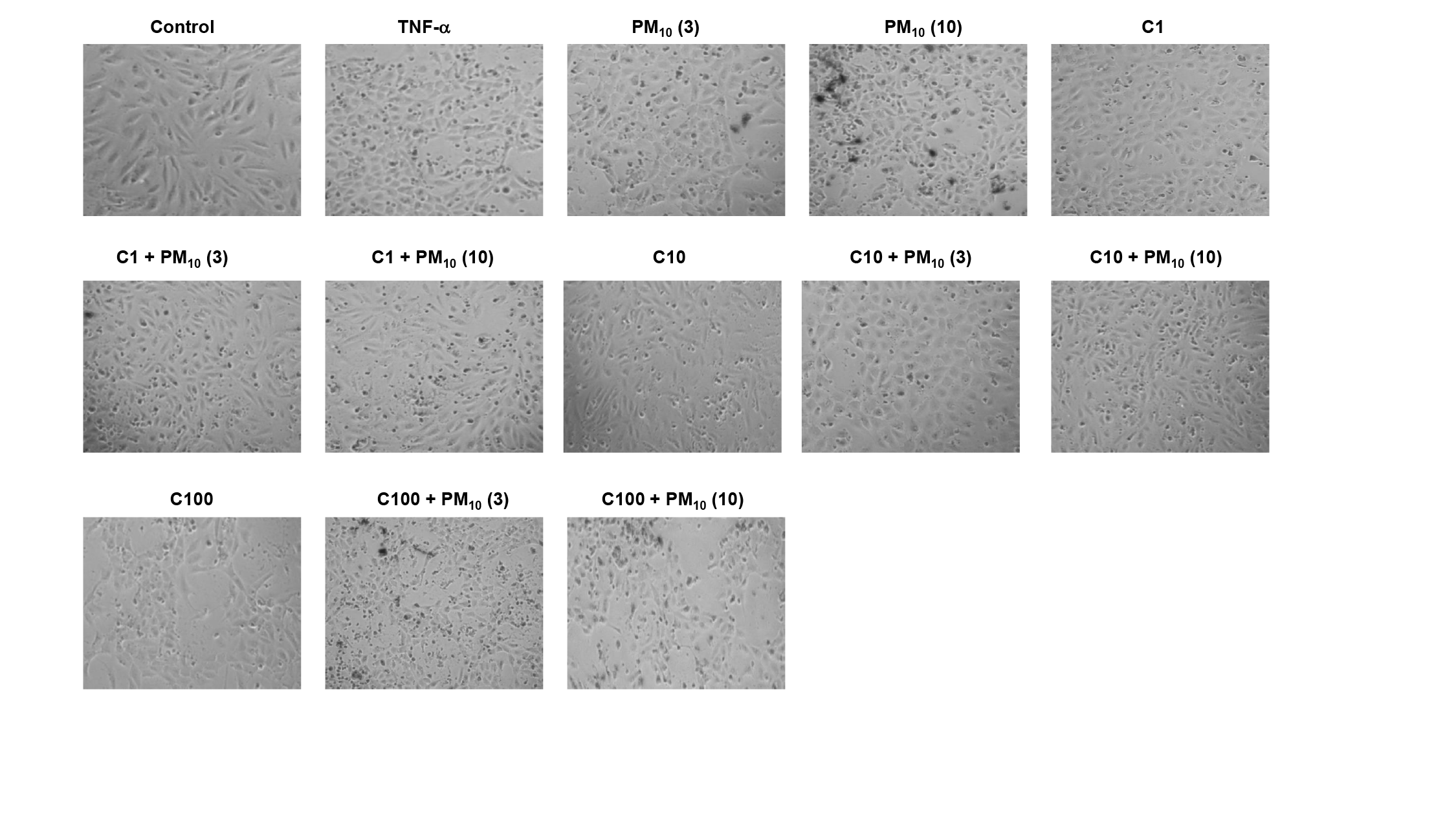

Supplement: S1 Fig — Cells were treated with curcumin at 1 (C1), 10 (C10) and 100 (C100) μM alone or in combination with 3 and 10 μg/cm2 of PM10 (3) and (10) for 24 h. Curcumin was added 1 h before the addition of PM10. TNF-α (10 ng/mL) was used as positive control. Photographs were taken with an optical microscope at 10X magnification. (TIF) [file pone.0188169.s001.tif]

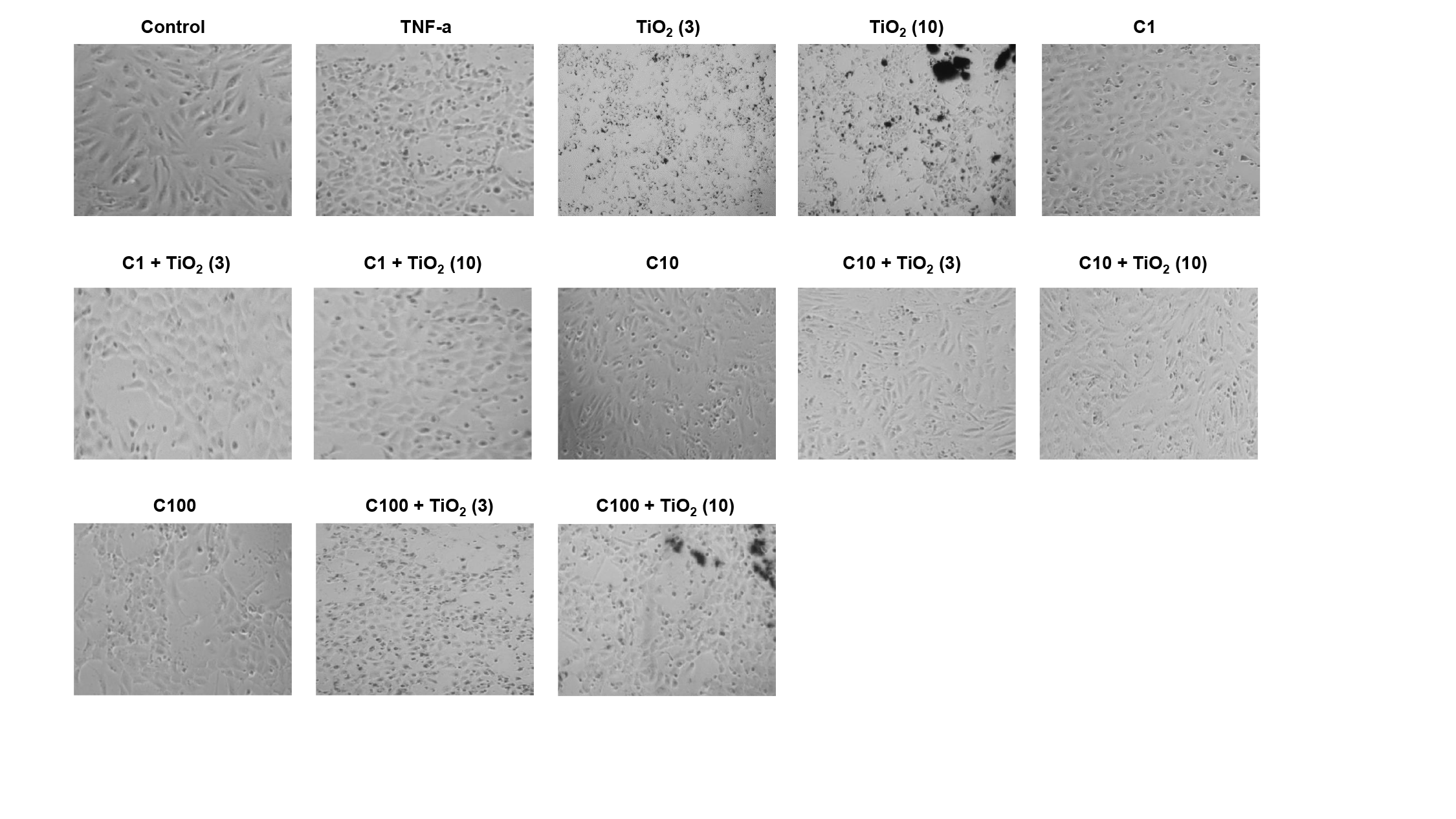

Supplement: S2 Fig — Cells were treated with curcumin at 1 (C1), 10 (C10) and 100 (C100) μM alone or in combination with 3 and 10 μg/cm2 of TiO2-NPs (3) and (10) for 24 h. Curcumin was added 1 h before the addition of TiO2-NPs. TNF-α (10 ng/mL) was used as positive control. Photographs were taken with an optical microscope at 10X magnification. (TIF) [file pone.0188169.s002.tif]

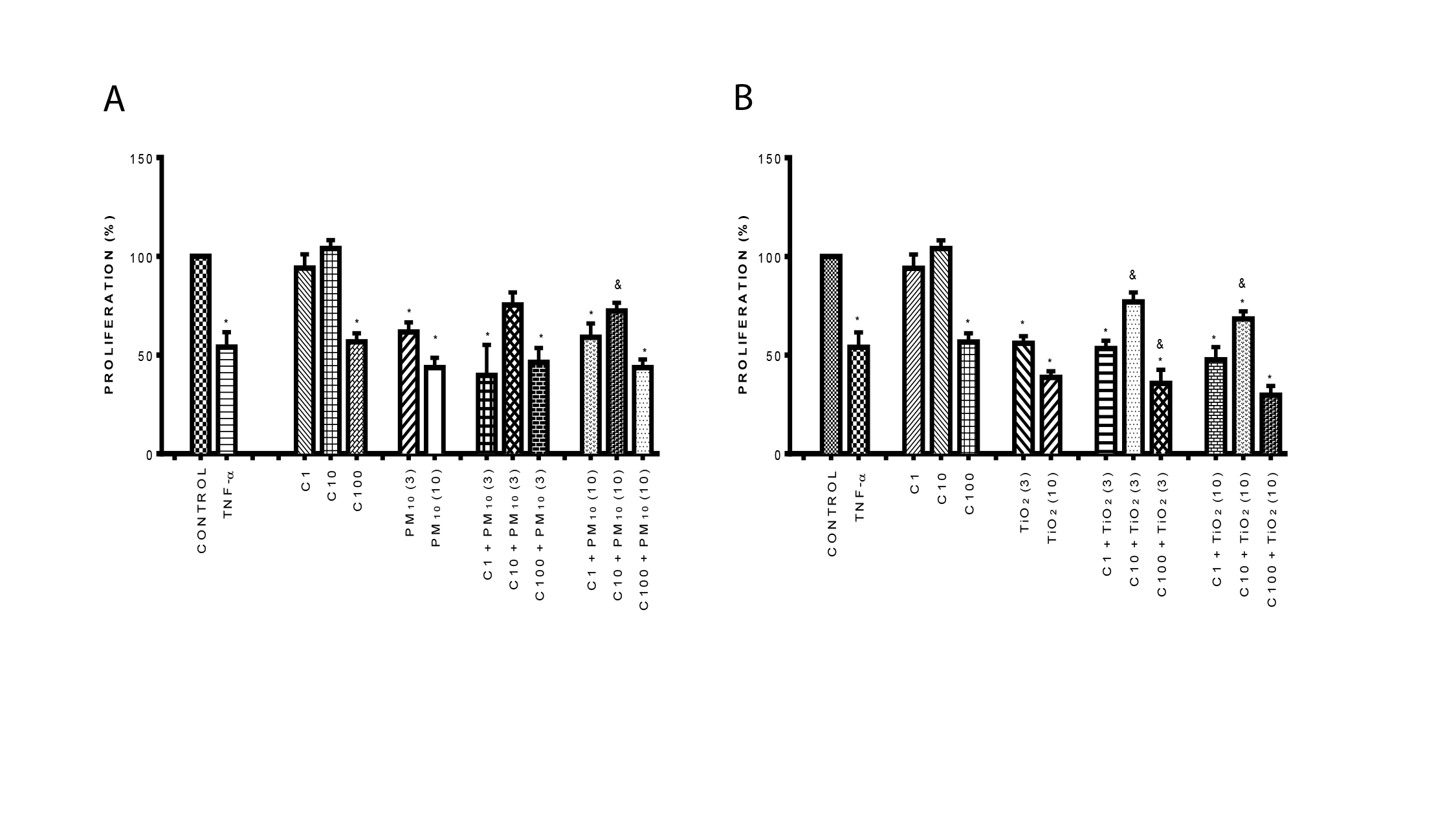

Supplement: S3 Fig — Cells were treated with curcumin at 1 (C1), 10 (C10) and 100 (C100) μM alone or in combination with 3 and 10 μg/cm2 of PM10 (3) and (10) (A), and with 3 and 10 μg/cm2 of TiO2-NPs (3) and (10) (B) for 24 h. Proliferation was evaluated with crystal violet staining. Curcumin was added 1 h before the addition of PM10 and TiO2-NPs. TNF-α (10 ng/mL) was used as positive control. Data show the mean ± standard deviation (SD) of three separate experiments, expressed as percentage of proliferation compared to control (100%). p < 0.05, experiments compared with untreated cells (Control) (*) and with PM10 or TiO2-NPs alone (&). (TIF) [file pone.0188169.s003.tif]
